# Effect of Time-Restricted Eating and Resistance Training on High-Speed Strength and Body Composition

**DOI:** 10.3390/nu15020285

**Published:** 2023-01-06

**Authors:** Joana M. Correia, Paulo D. G. Santos, Pedro Pezarat-Correia, Cláudia S. Minderico, Jorge Infante, Goncalo V. Mendonca

**Affiliations:** 1 Neuromuscular Research Lab, Faculdade de Motricidade Humana, Universidade de Lisboa, Estrada da Costa, 1499-002 Cruz Quebrada, Portugal; 2 CIPER, Faculdade de Motricidade Humana, Universidade de Lisboa, Estrada da Costa, 1499-002 Cruz Quebrada, Portugal

**Keywords:** intermittent fasting, exercise, strength, fat mass

## Abstract

This study examined the effects of four weeks of resistance training combined with time-restricted eating (TRE) vs. habitual diet on fat and fat-free mass as well as maximum and explosive force production in healthy, trained participants (18 males, aged 23.7 ± 2.6 years). The order of dieting was randomized and counterbalanced, and the participants served as their own controls. TRE involved an 8-h eating window and non-TRE involved a habitual meal pattern. Participants completed performance strength tests and body composition scans at baseline and post-intervention. The participants followed a structured training routine during each dietary intervention (four sets of maximum repetitions at 85% 1RM in five dynamic exercises, three times/week). Both interventions elicited deceases in fat mass (*p* < 0.05) but not in fat-free mass. After training (controlling for baseline values as covariates), non-TRE was compatible with better lower body jump performance than TRE (*p* < 0.05). Conversely, training with TRE elicited higher values in terms of peak force and dynamic strength index at the level of the upper body (*p* < 0.05). Thus, it can be concluded that there were no differences in fat mass and fat-free mass changes between interventions in already trained young males. Additionally, while the combination of TRE and resistance training might be beneficial for individuals focusing on developing high-speed strength performance at the upper body level, this is not applicable to those focusing on training the lower body.

## 1. Introduction

Increasing muscle mass and decreasing fat mass is a common, non-pharmacological approach for eliciting positive health outcomes and augmenting athletic performance [1]. Many people aiming at modifying body composition combine dietary strategies with regular exercise [1]. Intermittent fasting has gained popularity over the years due to its potential health benefits at the level of body composition, lipidemia, glycemia, insulinemia, blood pressure and inflammation [2,3,4,5]. Intermittent fasting is distinct from continuous energy restriction, in which daily energy intake is chronically reduced by up to 40%, irrespectively of meal frequency [6]. Moreover, certain intermittent fasting modalities have also been shown to produce higher compliance than continuous energy restriction, and this may be particularly relevant for long-term adherence [4]. There are several forms of intermittent fasting, including fasting for multiples hours, days or weeks [7]. All these protocols involve fasting periods that extend well beyond the duration of an overnight fast (usually for 12 h or more) and accommodate limited feeding time-windows, either with or without complementary energy restriction [7]. One particular form of intermittent fasting that has gained considerable popularity is time-restricted eating (TRE), which encompasses recurrent fasting periods on a daily basis, ranging from 12 to 21 h, followed by ad libitum food and fluid intake for the remaining hours of the day [8]. Two common TRE variations involve a daily 16-h fast with an 8-h feeding window or a daily 20-h fast with a 4-h feeding window [8]. Unfortunately, only a small number of studies have examined the effectiveness of TRE on decreasing adiposity while maintaining muscle mass and strength [1,9,10]. In these studies, despite leading to a state of reduced energy intake, the combination of TRE with short-term resistance training exerted no adverse impact on lean mass retention or muscular improvements in young healthy males [1,10]. Specifically, while Moro et al. found no differences between TRE and a control diet in terms of increasing the leg-press one-repetition maximum (1RM) [10], Tinsley et al. observed that adaptations at the level of muscular performance were not negatively affected by the TRE group [1]. In fact, these authors reported significant enhancements in terms of lower body muscular strength and endurance as well as upper body muscle endurance [1]. In another study, the combination of TRE with resistance training resulted in similar muscular gains over time as seen with a control diet [9]. Ultimately, all these findings are well aligned with the concept that resistance training minimizes the loss of skeletal muscle mass and enables the maintenance of muscular strength during periods of energy restriction [11].

Past research examining the combined effect of TRE and resistance training on muscle performance has focused primarily on adaptations at the level of the muscle cross-sectional area and slow-speed strength (i.e., 1RM) [1,10]. To our knowledge, the impact of this specific dietary approach on high-speed strength (e.g., explosive strength) has not yet been examined. High-speed strength represents a key factor of performance during movements characterized by reduced contractions times (i.e., duration < 250 ms) and involving an explosive type of muscle action (e.g., sprinting, jumping or kicking) [11,12]. There is compelling evidence that short-term resistance training (e.g., four weeks) is effective for enhancing high-speed strength via neural and mechanical adaptations [13]. Yet, whether TRE affects the overall positive impact of resistance training on high-speed strength is presently unknown. For this reason, using a randomized crossover design, this study examined the effects of short-term resistance training combined with TRE on the maximum as well as explosive force production of healthy, trained males. Additionally, the overall effects of this intervention on body composition in these individuals were also determined. It was hypothesized that, when compared to a habitual dietary pattern, TRE would lead to greater fat-mass loss (while preserving fat-free mass) without compromising performance.

## 2. Materials and Methods

### 2.1. Participants

Eighteen male physical education students, well accustomed to resistance training, with normal blood pressure (all ≤120/80 mmHg) and not taking any medication, were initially enrolled in this crossover trial [14]. All participants were recruited from the Faculty’s surroundings via word of mouth, and they all volunteered for inclusion in this experimental design. Inclusion was limited to healthy males with previous experience in resistance training (performing more than three sessions/week on a consistent basis, over the past three years) [15]. For homogeneity purposes, the following inclusionary criteria were also defined: (1) ratio of bench press 1RM/body mass > 1.26 (>80 percentile for 20–29 years old males) and (2) ratio of leg-press 1RM/body mass > 2.08 (>80 percentile for 20 to 29 years old males) [16]. In addition, only participants with no conditions that could be aggravated by the study procedures were included (i.e., non-smokers, normotensive, normal-blood lipid and glucose profile, non-overweight, non-medicated, free of any known disease as assessed by medical history and orthopedic issues limiting exercise performance) [17,18].

Each participant was requested to avoid heavy exercise for at least 24 h before each testing session and to have nothing to eat from midnight until the testing session on the subsequent morning. Participants were also asked to refrain from caffeine ingestion and to empty their bladders before testing. Participants were fully informed of the purposes, risks and discomfort associated with the experiment before providing written, informed consent. All participants read and signed an informed consent document with a description of the testing procedures included in the study, which was carried out with the approval from the University’s Institutional Review Board (CEFMH N°12/2018) and in accordance with the Declaration of Helsinki.

### 2.2. Experimental Design

**Figure 1:**
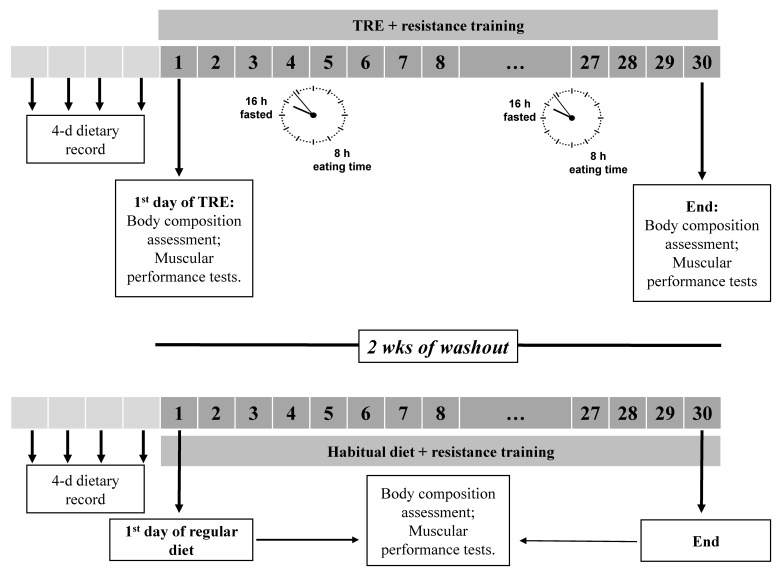
Schematic representation of the study protocol. The experimental design consisted of two dietary interventions involving four weeks of time-restricted eating (TRE) and non-time-restricted eating (non-TRE). TRE interventions followed a 16/8 time-restricted feeding protocol. The order of dieting was randomized and counterbalanced, and two weeks of washout separated both conditions. Baseline dietary records were obtained prior to each dietary intervention. Body composition assessments and muscular performance tests were performed in consecutive days at baseline and after four weeks of intervention under both conditions. The participants were required to follow a structured training routine during each dietary intervention (three weekly sessions of four sets of 8–10 repetitions at 85% of the 1-RM leg press, bench press, leg extension, leg curl, shoulder press and lat pulldown).

The experimental design of the present study is depicted in Figure 1. All participants were tested over the course of several different visits, at approximately the same time of day (between 06.00 and 08.00 a.m.). Measurements were taken before and after thirty days of each dietary intervention (TRE vs. habitual diet), combined with a structured and supervised regimen of resistance training. Participants were familiarized with the experimental protocol one week before testing. Familiarization consisted of one session designed to ensure adaptation to all testing procedures. Blood pressure was also measured at the beginning of these sessions. All assessments were performed on the same day and the order of testing was as follows (the average session time was approximately 150 min): (1) assessment of body composition and (2) performance strength assessments (i.e., lower body: countermovement jump, squat jump and mid-thigh pull; upper body: isometric bench press and the bench press throw). Participants served as their own controls, and the order of dieting was randomized, using a computer-generated algorithm, and counterbalanced (i.e., at study entry, nine participants were randomly allocated first to the TRE and another nine to the non-TRE dietary intervention first).

Prior to each dietary intervention, participants were asked to fill out a dietary record over four consecutive days. As in previous studies, a two-week washout period (participants followed no specific diet and exercise was not allowed) separated both conditions [19]. The experimental design consisted of two dietary interventions: (1) 30-days of TRE and (2) 30-days of habitual diet (non-TRE). The final testing sessions (post-30 days of each intervention) were completed between 72–96 h after the last training session.

### 2.3. Body Composition

Body mass was measured with the participants wearing lightweight clothes and no shoes. Body mass was measured to the nearest 0.1 kg using a calibrated digital scale (TANITA^®^ BF-350 scale, Arlington Heights, IL, USA). Height was also measured to the nearest 0.1 cm using a stadiometer at the first testing session (standing digital scale/height rod attached). Subsequently, each participant was scanned with dual-energy X-ray absorptiometry (total body scan) to estimate fat mass, fat-free mass and % body fat (fan-beam mode, software version 5.67, enhanced whole body analysis, Hologic Explorer-W, Waltham, MA, USA). Skeletal muscle mass was also estimated using the following formula: skeletal muscle mass (kg) = 1.19 × appendicular lean soft tissue (kg) − 1.65 [20]. Based on test–retest measures including 10 participants (other than the ones included in this study), the coefficient of variation for fat mass and fat-free mass in our laboratory is 1.7 and 0.8%, respectively. The intraclass correlation coefficients (ICCs) obtained for these respective variables were 0.997 and 0.999.

### 2.4. Strength Performance Assessment

The participants visited our laboratory to perform all strength tests on the same day. Testing began with a 5 min warm-up period using a self-selected pace on a cycle ergometer (Monark Ergomedic 828E, Varberg, Sweden), as described elsewhere [1]. This warm-up period was followed by the assessment of countermovement jump and squat jump performance. After performing the lower body ballistic strength tests, the participants completed the mid-thigh pull test. After concluding the lower body strength assessments, the participants were tested for upper body muscle strength. In accordance, all participants began these assessments by performing the isometric bench press test. Then, after completing this test, the participants performed the bench press throw test. The lower body strength assessments were performed using a force plate (Bertec, Colombia, SC, USA) connected to an external analog-to-digital converter (MP100–Biopac™ Systems, 16-bits) at a sample rate of 1000 Hz. The signal was low-pass filtered at 10 Hz (zero phase shift 4th order Butterworth filter) and data processing was carried out using AcqKnowlege 3.9.1 software (Biopac Systems, Inc., Goleta, CA, USA). The assessments of upper body muscle strength were performed using a bench incorporating four force sensors in a force plate placed at the bench base (Flintec Beam Load Cell BK2-200 kg, Hudson, MA, USA). The summed vertical ground reaction force was obtained at a sample rate of 1000 Hz and low-pass filtered at 10 Hz (zero phase shift 4th order Butterworth filter). The force plate was connected to the same external analog-to-digital converter as described for the lower body assessments. The reliability and validity of this specific setup for analyzing the characteristics of the force–time curve during upper body exercise have been published elsewhere [21,22].

### 2.5. Lower Body Explosive and Reactive Strength (Countermovement and Squat Jump)

Vertical jump height was measured with the countermovement jump and the squat jump. Once standing on the force plate, the participants performed two submaximal practice jumps for both the countermovement jump and the squat jump. One member of the research team demonstrated both techniques to each participant at the beginning of each experimental trial. Then, after two minutes of passive rest, the participants completed three countermovement maximal jumps, separated by another two minutes of rest between attempts [23]. For the countermovement jump, all participants were instructed to assume an erect standing position and then perform a crouching action, immediately followed by a maximal jump (as high as possible). Participants were instructed to hold their hands at the waist level during each attempt to eliminate any influence in terms of arm swing on performance. They were also asked to jump with maximum effort to achieve the highest vertical displacement possible [24]. After five minutes of recovery from the last countermovement jump attempt, the participants performed three squat jumps interspersed by two minutes of passive recovery. The squat jump technique required that each participant started from the squat hold position for at least 1 s (thighs parallel to the floor), before jumping vertically with the intent of reaching maximum height without prior countermovement [23]. All squat jumps were performed with the participants’ hands placed at the waist level throughout the full range of take-off, flight and landing movements. The squat jump and countermovement jump are reliable and valid measures for the estimation of lower body explosive power in physically active males (the coefficient of variation for the squat jump and countermovement jump height are 6.5% (ICC: 0.98) [25] and 13.9% (ICC: 0.93), respectively [26]) and for the estimation of peak force (the coefficient of variation for the squat jump and countermovement jump are 4.3 (ICC:0.97) and 3.5% (ICC:0.97), respectively [27,28]).

### 2.6. Lower Body Maximal Voluntary Contraction (Mid-Thigh Pull)

Lower body maximal voluntary contraction (MVC) was measured using the isometric mid-thigh pull. Testing was performed with the participants standing on the force plate within of a custom-made power rack (anchored to the floor) that enabled the bar to be fixed at different heights (i.e., adjustable by ±4 cm). The bar height was individualized to each participant based on their self-selected mid-thigh clean position [29]. As in previous studies, the participants were asked to grip the bar (closed, regular grip) and position their hands and feet shoulder width apart, with each knee flexed to an angle of ~140°, the hip angle at ~125° and the trunk upright [30,31]. The knee and hip angles of all participants were measured with a goniometer (Lafayette Instrument, Model 01135, Lafayette, IN, USA) to ensure high reproducibility between trials and time points. Three attempts were performed by each participant, with a contraction duration of 5 s and 1 min of rest [30]. Verbal encouragement was provided to all participant, as they were asked to pull as fast and strong as possible during each attempt [30]. Based on the test–retest measures of a pilot study including eight participants (other than those included in this study), the coefficient of variation in peak force, maximum rate of force development (RFD), Force _50 ms_, Force _100 ms,_ Force _150 ms_ and Force _200 ms_ for this specific mid-thigh pull protocol corresponds to 2.3, 8.1, 20.1, 10.9, 7.9 and 6.4%, respectively. The ICCs obtained for these variables were 0.987, 0.816, 0.773, 0.907, 0.923 and 0.893, respectively.

### 2.7. Upper Body Maximal Voluntary Contraction (Isometric Bench Press)

The upper body MVC was measured with the bench press exercise performed on a Smith machine (Matrix Fitness^®^ GI Smith machine, Cottage Grove, WI, USA). Participants assumed the supine position and all attempts were completed while respecting five points of contact (i.e., the firm planting of their feet, gluteus maximus, back and head), beginning with their elbows flexed at 90° and them gripping the bar aligned with the individual nipple line [32]. The hand position on the bar was defined as the distance measured between elbows at 90° shoulder abduction. The hand distance was recorded for each participant to ensure reproducibility at all testing time points. Participants were given a countdown before each MVC, and specific verbal encouragement was provided throughout each attempt. Three attempts were performed by each participant, with a contraction duration of 5 s and 1 min of rest [32]. Based on test–retest measures (as described above), the coefficient of variation for this specific protocol, namely peak force, maximum RFD, Force _50 ms_, Force _100 ms,_ Force _150 ms_ and Force _200 ms_ is 5.8, 11.2, 19.8, 11.1, 8.1 and 6.0%, respectively. The ICCs obtained for these variables were 0.952, 0.837, 0.714, 0.756, 0.499 and 0.719, respectively.

### 2.8. Upper Body Explosive and Reactive Strength (Bench Press Throw)

The bench press throw test was performed on a Smith machine using a load of 45% 1RM (based on pre-intervention bench press 1RM, as explained below). According to previous research, this load maximizes the power output attained by athletes during the execution of this particular exercise [33,34]. Participants were instructed to grasp the bar and lift it off the safety stops of the Smith machine (the hand distance was set similarly to that described above). The bar was then lowered eccentrically in a slow controlled manner. The eccentric phase terminated at a bar travel distance compatible with reaching 90° elbow flexion (~2–3 cm above the nipple line). Each participant was instructed to exert maximal concentric force as rapidly as possible as soon as the bar touched the Smith machine’s safety stops. This was done to take advantage of the stretch-shortening muscle cycle [35]. All participants performed three attempts of one repetition with 45% 1RM and 4 min of rest were allowed between sets [36]. The force–time curve was monitored online throughout each attempt. Based on test–retest measures (as described above), the coefficient of variation and ICC for the peak force obtained with the bench press throw exercise is 9.3% and 0.921, respectively.

### 2.9. Data Analysis of Muscle Performance

The force–time variables computed from the countermovement jump and squat jump included jump height and peak force (the highest force value reached during each ballistic test). In addition to these variables, for the bench press throw test, the fastest mean concentric velocity of the bar was also recorded. For the isometric MVC tests, the peak force was determined as the highest force value reached during each mid-thigh pull and bench press attempt. The maximum RFD was additionally analyzed for each attempt as the highest peak force–time curve slope using 20-ms time windows [37]. Explosive force was defined as the %MVC attained at specific time points (50, 100, 150 and 200 ms). This approach represents a relative measure of explosive force production and translates the ability to recruit the individual force reserve over time [12]. The onset of force development (start of contraction) was defined as the time point at which the force curve exceeded the average baseline values by 3 N [12] for all isometric MVC attempts. Contractions with evidence of countermovement were discarded from the analyses. The peak force (for the dynamic tests) and maximum RFD (for dynamic and isometric tests) were measured in absolute and normalized terms relative to isometric MVC. The dynamic strength index was calculated to detect specific deficiencies in producing dynamic and explosive force. This index was expressed as the ratio of the ballistic to isometric peak force (i.e., dynamic strength index = ballistic peak force/isometric peak force) [33]. This reflects the extent to which an individual is able to apply force dynamically in relation to his maximum isometric force production [38]. The participants’ dynamic strength indexes for the lower body were calculated using the peak values for the countermovement jump and squat jump peak force and isometric mid-thigh pull peak force, and the participants’ dynamic strength indexes for the upper body were calculated using the peak values for the bench press throw peak force and bench press peak force. For statistical purposes, the repetition with the highest maximum RFD value was used in these analyses as long as the peak force value was >95% of the highest peak force value achieved in the three repetitions.

Vertical jump height was calculated based on the approximation of kinematic laws by measuring the individual flight time. The flight time was measured with an electronic apparatus (Chronojump Boscosystems, Barcelona, Spain). The Chronojump consists of a digital timer (+0.001 s) connected by a cable to a resistive (or capacitive) platform. The timer is triggered by the participant’s take off from the platform and is stopped at the moment of touch down [36]. The displacement of the center of gravity during contact can be estimated assuming that the vertical velocity from the lowest point of the center of gravity to the release increases linearly [36]. The vertical jump height (in countermovement jump and squat jump) was calculated using the impulse–momentum relation and expressed in centimeters [36]. The peak force was calculated as the maximum force achieved over the force–time curve during each jump [28]. Only the attempts compatible with best result were used for posterior analysis.

A previously validated linear position transducer (Chronojump Boscosystems, Barcelona, Spain) was used to measure movement velocity during the bench press throw tests [39]. The mean and the peak concentric velocities were recorded in all attempts. Then, the mean power achieved during the concentric phase of each attempt was additionally calculated. All data were analyzed using a code written in Matlab^®^ R2014a (The MathWorks, Natick, MA, USA).

### 2.10. Dietary Intake

One week before testing (pre-TRE and non-TRE), participants were asked to fill out a dietary record over four consecutive days to estimate energy intake, macronutrient distribution and the timing of food ingestion. The Food Processor^®^ Nutrition Analysis software (ESHA, Salem, Oregon) was used to compute the individual daily dietary intake, namely the total energy intake and carbohydrate, lipid and protein content. Following the collection of dietary data, participants were instructed to maintain their habitual food preferences over the course of the study to minimize noncompliance. TRE interventions followed a 16/8 protocol [10]. During the non-TRE condition, participants consumed 100% of their energy needs and were instructed to continue with their habitual dietary patterns for the duration of the intervention without any timing restrictions. Conversely, during the TRE condition, participants consumed two to three meals (ad libitum and without ER) within an 8-h window (between 1 and 9 p.m.). Only water, tea and coffee (without caloric additives) were permitted to be consumed in the remaining 16 h per 24-h time.

### 2.11. One-Repetition Maximum Testing

One week before initiating each dietetic protocol, strength assessments were performed to directly obtain the 1RM for all exercises included in the training program. The bench press 1RM was also measured to allow the subsequent calculation of the individual load to be used when testing the bench press throw performance (45% 1RM). Testing procedures followed the recommendations advanced by the American Society of Exercise Physiologists [40]. Briefly, the protocol was initiated with 8–10 repetitions performed at a load of ~50% of the estimated 1RM. Then, the load was adjusted to 75% of the estimated 1RM and each participant was instructed to complete five repetitions. These two initial sets of muscle contractions served as specific warm-up. Subsequently, the load was adjusted as needed and each participant performed the maximum number of repetitions to volitional failure (with verbal encouragement). The load was increased by 5% whenever participants were able to complete more than two repetitions before failure. Three minutes of rest were allowed between the first three attempts. Additional tests required 5 min of rest to ensure full recovery. The 1RM was accepted as the maximum load that each participant could lift in a single maximum dynamic muscle contraction using the full range of motion [41].

### 2.12. Training Program

Training was standardized between conditions and consisted of three weekly sessions, performed on Monday, Wednesday and Friday, for four weeks (i.e., 12 exercise sessions). A member of the research team supervised the first training session of each week. Participants were instructed not to perform any other type of training during the course of their participation in this study. The training protocol involved four sets of maximum repetitions at 85% 1RM, with 90–120 s of rest between sets and exercises [42]. The concentric phase of each lift in a fast-controlled fashion and the eccentric phase was lasted ~1 s. A pause of 1 s was additionally imposed between phases. During the first two weeks, participants trained at 85% of the initial 1RM in all sets for each exercise. Then, whenever the participants were able to perform between two extra repetitions in the first set, they were encouraged to increase their upper and lower body exercise loads by 5 and 10%, respectively [43]. The exercise prescription involved a whole body routine, including five dynamic exercises completed in the following order: (1) leg press, (2) chest press, (3) leg curl, (3) lat pull down, (4) leg extension and (5) shoulder press. In each session, the participants also performed four sets of callisthenic abdominal crunches (85% of the maximum number of repetitions performed by each participant on the first training session of each week). Participants were asked to record and return weekly session logs to the principal investigator (including detailed information on the volume load per exercise). All participants attended 100% of the training sessions and, as in previous studies, exercise sessions were completed within the feeding window period [10].

### 2.13. Statistical Analysis

Before comparing both conditions (TRE vs. non-TRE), data were tested for normality and homoscedasticity with the Kolmogorov–Smirnov and Mauchly’s test, respectively. Based on the results from a previous study [1], absolute lower body strength gains correspond to 77 ± 31.3 and 39 ± 36.5 kg (mean difference and standard deviation of the difference) after four weeks of resistance training combined with TRE and non-TRE, respectively. Thus, a sample size of 14 participants per group was estimated to have 80% power, correctly rejecting the null hypothesis. The sample size was expanded to 18 participants to accommodate an attrition rate of ~30% [44]. Therefore, 18 young participants were considered eligible for inclusion in this study.

A one-way repeated measures ANOVA was used to determine baseline differences between conditions in terms of impact on strength and body composition. To analyze the differences between conditions after each intervention, an ANCOVA was computed for each dependent variable, entering the condition as a within factor and the baseline measures as covariates. To determine the effect of each diet on the evolution of the dependent variables from pre- to post-training, an ANOVA with repeated measures was performed (condition (TRE vs. non-TRE) by time (pre- vs. post-intervention)). When a significant interaction was detected, *t* tests were used for post hoc comparisons. Adjustment for multiple comparisons were made by using Bonferroni’s correction. The partial eta-squared values (proportion of total variance that is attributable to an effect) were reported to indicate effect sizes (ES) (small, medium and large effect: 0.01, 0.06 and 0.14, respectively) [45]. Statistical analyses were performed using Statistical Package for the Social Sciences (version 25.0, SPSS Inc., Chicago, IL, USA). All data are reported as mean ± SD. Statistical significance was set at *p* < 0.05.

## 3. Results

As shown in Table 1, the dietary energy intake and macronutrient distribution were similar at baseline between conditions (*p* > 0.05). Table 2, Table 3 and Table 4 depict the effects of TRE and habitual diet on body composition and on indices of muscle strength after four weeks of intervention (TRE vs. non-TRE). At baseline, there were no differences between conditions in terms of the participants’ body composition or muscle strength (Table 2, Table 3 and Table 4). With exception of the mid-thigh pull peak force, for which the pre-training value was lower under the TRE condition (F = 8.2, *p* = 0.01, ES = 0.33), no other main effects were detected for muscle strength before training (Table 3 and Table 4).

The repeated measures ANOVA showed that there were no differences between conditions in terms of a significant reduction in fat mass (time main effect: F = 5.1, *p* = 0.04, ES = 0.23) after four weeks of intervention (Table 2). As shown in Table 4, both interventions were effective for improving explosive upper body muscle strength at 100 (F = 8.0, *p* = 0.01, ES = 0.35), 150 (F = 8.1, *p* = 0.01, ES = 0.37) and 200 ms (F = 5.4, *p* = 0.04, ES = 0.29) of MVC from pre- to post-training. Finally, a condition-by-time interaction was detected for the bench press throw peak force (F = 10.3, *p* = 0.005, ES = 0.38) and for the bench press throw dynamic strength index (F = 14.6, *p* = 0.001, ES = 0.46). As depicted in Table 4, while training with TRE increased the peak force and dynamic strength index over time for the bench press throw, the opposite occurred with non-TRE.

After four weeks of intervention (controlling for baseline values as covariates), there was a significant condition main effect for select variables of lower (squat jump peak force: F = 7.2, *p* = 0.02, ES = 0.32; countermovement jump peak force: F = 4.7, *p* = 0.04, ES = 0.24; countermovement jump height: F = 6.0, *p* = 0.03, ES = 0.28) and upper body muscle strength (isometric bench press RFD_max_: F = 4.8, *p* = 0.05, ES = 0.24). All these variables attained greater values at the post-training time point under the non-TRE condition (Table 3 and Table 4). No other variable attained significant differences between conditions at the post-training time point.

## 4. Discussion

The present study indicates that, when combined with four weeks of structured resistance training, TRE is as effective as a habitual dietary pattern in decreasing fat mass while preserving fat-free and appendicular skeletal muscle mass in trained young males. Despite this, the effects of both interventions on upper and lower body strength were different. While after training, the habitual diet condition was compatible with a slightly greater dynamic high-speed strength performance in the lower body, this was not the case for upper body exercise. In fact, improvements in ballistic bench press performance were only seen after four weeks of TRE. In addition, these data also show that both interventions were similarly effective for improving explosive muscle strength in response to isometric upper limb MVC. Despite this, it should be noted that the magnitude of change in the maximum RFD obtained during isometric upper body MVC was greater under the non-TRE condition. Taken together, this study provides preliminary evidence that, when combined with structured resistance training, TRE might potentiate performance in dynamic tasks involving upper body high-speed strength, but this does not occur at the lower body level.

According to the current findings, both conditions were similarly effective in decreasing fat mass, without a parallel loss of fat-free or skeletal muscle mass. Specific biological mechanisms have been advocated to explain the effects of TRE on fat mass reduction (e.g., increased mitochondrial biogenesis, enhanced secretion of adiponectin, noradrenaline and growth hormone) [23]. It has even been contended that, when combined with resistance training, TRE displays an adjunctive role in preserving or delaying possible fat-free mass losses while promoting fat mass reductions [23]. Yet, here, it was shown that the combination of four weeks of TRE with resistance training was not sufficient to induce further improvements in body composition compared to that seen following resistance training accompanied by a habitual dietary pattern. Thus, it can be concluded that resistance training per se, instead of diet, had the most influential impact on decreasing fat mass over four weeks of intervention. It is well known that resistance training can chronically influence resting energy expenditure [46]. In addition, acute resistance training also inflates excess post-exercise oxygen consumption and shifts the respiratory exchange ratio towards values compatible with greater fat utilization [46]. Ultimately, all these factors play an important role in decreasing fat mass after resistance training, independently of dietary pattern.

It was observed that neither intervention was effective for improving peak or explosive force in response to isometric mid-thigh pull after training. This is in line with certain past studies showing that, due to its specificity, dynamic resistance training has no impact on improving isometric strength performance [47]. Even though both conditions were compatible with a similar jump height and peak force in response to the countermovement jump at the pre-training time point, this was not the case after four weeks of intervention. Specifically, it was shown that the combination of resistance training and a habitual dietary pattern resulted in greater values for both of these variables after training (~1.5 and 2.3% for jump height and peak force, respectively). Despite having achieved statistical significance, it is relevant to note that the magnitude of these differences falls within the coefficient of variation obtained for both variables (~14 and 3.5% for jump height and peak force, respectively). This indicates that the practical relevance of such differences is questionable and should not be interpreted as an unequivocal sign of enhanced performance.

Similar to the case of the countermovement jump, no differences between conditions were observed for the peak force of the squat jump before training. Again, after four weeks of intervention, the combination of resistance training and a habitual dietary pattern resulted in a greater peak force in the squat jump (~6%). The magnitude of this difference is greater than the coefficient of variation reported for this specific variable (~4.3%) [27]. Therefore, these findings indicate an effective benefit of a habitual diet over TRE for improving this specific variable in the context of resistance training (instead of reflecting a “natural” between-day fluctuation in terms of peak force in the squat jump). It is relevant to reinforce that these gains in high-speed strength performance at the post-training time point were not accompanied by differential gains in appendicular skeletal muscle mass between conditions. Ultimately, this suggests that they may be secondary to neural adaptations within the neuromuscular system [13].

Upper body isometric strength performance was similar between conditions before training. In contrast, after four weeks of intervention, there were greater values in terms of peak RFD (obtained during MVC) under the condition combining resistance training and a habitual dietary pattern (~9%). An elevated peak RFD enables enhanced force production during rapid movements, and this is important for both athletic as well as nonathletic populations [11]. This is particularly relevant in circumstances when force production times are short (e.g., 100–300 ms), such as when reversing a fall or in various athletic events (e.g., sprints, throws and jumps) [11]. There is compelling evidence that, after a period of resistance training, RFD increases both in the early and later phases of force–time curve [11]. However, the absolute reliability of the peak RFD of the isometric bench press is relatively poor (coefficient of variation of ~11%), and the magnitude of the differences between both conditions obtained in the present study does not allow us to conclude that there was a true difference between treatments.

In contrast to the results obtained for peak RFD, there were significant improvements, from pre- to post-training, in explosive force production from 100 to 200 ms in terms of bench press isometric MVC under both conditions. This indicates that qualitative changes may have occurred with resistance training, potentially involving alterations in motoneuron recruitment, rate coding, discharge doublets, myosin heavy chain isoform expression and sarcoplasmic reticulum calcium kinetics [11]. The magnitude of improvement in explosive force was expressive (TRE: 9.1–12.9% and non-TRE: 5.9–7.1%) and similar with both interventions. In accordance, it can be concluded that resistance training was the primary trigger for this specific adaptation, independently of either prescribed diet. However, it should be noted that such enhancements only exceeded the coefficient of variation inherent to explosive force obtained at 100 and 200 ms (10.9 and 6.4%).

Both conditions were compatible with similar values in terms of upper body high-speed strength performance at pre-training. Yet, while the combination of resistance training with a habitual dietary pattern resulted in a significant reduction (−15%) in peak force in response to the bench press throw exercise after training, this was not seen with TRE. The magnitude of reduction in this specific variable following the intervention involving a habitual dietary pattern was greater than its coefficient of variation (~9.3%). Hence, while TRE induced a slight increase in upper body high-speed strength performance over time, the combination of resistance training with a habitual dietary pattern resulted in its decrease after four weeks of intervention. This finding agrees with isolated scientific data involving the interaction of certain resistance training regimens and high-speed strength performance [48]. The dynamic strength index followed the same adaptive trajectory (i.e., a slight enhancement with TRE and a reduction of 16% with a habitual dietary pattern) because it represents the ratio between the individual ballistic and isometric peak force [49]. The dynamic strength index provides relevant information concerning how strong the individual is and how much of that strength can be used during dynamic high-speed movements [49]. Greater dynamic strength index values are associated with an enhanced ability to perform general sports skills, such as jumping, sprinting and directional changes [50]. A heightened dynamic strength index has also been associated with an overall improvement in motor performance and reduced risk of injury [50]. However, it should be emphasized that, since both these variables (the peak force and dynamic strength index of the thrown bench press exercise) remained similar between conditions at the post-training time point, the practical relevance of our findings in the context of sports training is most likely weak.

### 4.1. Limitations

Certain limitations of the present study should be taken into account. First, energy and macronutrient intake were estimated based on self-reports using dietary records over four consecutive days. This approach has known limitations in terms of the accurate assessment of nutritional intake [10]. Second, dietary records were not obtained during the course of each intervention. Therefore, it is not possible to determine a cause–effect relationship between our findings and fluctuations in energy intake over time with each intervention. Third, only young male physical education students well accustomed to strength training were included. For this reason, these results cannot be generalized to elite athletes, females or older adults.

### 4.2. Conclusions

Based on the findings of this study, it can be concluded that four weeks of TRE combined with resistance training is not sufficient to elicit additional changes in fat mass and fat-free mass in already trained young males (vs. non-TRE). Conversely, the combination of TRE with resistance training was shown to exert a slight beneficial effect in response to upper body tasks involving high-speed dynamic strength performance. Despite this, it should be noted that, when compared to that seen with a habitual dietary pattern, TRE displayed a negative impact on high-speed strength performance at the level of the lower body. Taken together, this study provides preliminary evidence that while the combination of TRE and resistance training might be beneficial for individuals focusing on developing high-speed strength performance at the upper body level, this is not applicable to those focusing on training the lower body.

## Figures and Tables

**Table 1:** Estimated daily dietary intake obtained on four days immediately before time-restricted eating (TRE) and habitual diet (non-TRE).

Variables	Before TRE	Before Non-TRE	*p* Value
Energy intake (kcal)	2433.3 ± 760.5 (2055.1–2811.5)	2427.0 ± 556.8 (2150.1–2703.9)	0.96
Carbohydrate (%)	44.3 ± 7.1 (40.8–47.9)	44.1 ± 6.0 (41.1–47.0)	0.87
Fat (%)	31.6 ± 5.5 (28.8–34.3)	31.0 ± 4.8 (28.6–33.5)	0.70
Protein (%)	23.3 ± 3.8 (18.5–30.9)	24.3 ± 3.5 (18.9–30.4)	0.25
Protein (g/kg)	1.9 ± 0.6 (1.0–2.8)	2.0 ± 0.5 (1.1–2.9)	0.45

Values are mean ± SD and 95% confidence interval.

**Table 2:** Changes in body composition after four weeks of time-restricted eating (TRE) and habitual diet (non-TRE).

	TRE		Non-TRE	
	Pre-Intervention	Post-Intervention	Pre-Intervention	Post-Intervention
Body composition				
Body mass (kg)	73.2 ± 7.2 (69.6–76.8)	72.8 ± 7.1 (69.3–76.3)	73.0 ± 6.9 (69.6–76.4)	73.1 ± 7.0 (69.6–76.6)
Fat mass (kg) *	12.8 ± 4.4 (10.6–15.0)	12.2 ± 4.6 (9.9–14.5)	12.7 ± 4.2 (10.6–14.8)	12.5 ± 4.5 (10.2–14.7)
Fat-free mass (kg)	59.4 ± 4.6 (57.1–61.7)	59.6 ± 4.6 (57.4–61.9)	59.2 ± 4.8 (56.8–61.6)	59.6 ± 4.5 (57.4–61.8)
Skeletal muscle (kg)	28.6 ± 2.8 (27.2–29.9)	28.6 ± 3.0 (27.1–30.1)	28.6 ± 3.2 (27.1–30.2)	28.8 ± 2.9 (27.4–30.3)

Values are mean ± SD and 95% confidence intervals. * Time main effect (ANOVA: *p* = 0.04 from pre- to post-intervention).

**Table 3:** Changes in indices of muscle strength (lower limb) after 30 days of time-restricted eating (TRE) and habitual diet (non-TRE).

		TRE		Non-TRE	
		Pre-Intervention	Post-Intervention	Pre-Intervention	Post-Intervention
Isometric mid-thigh pull	Peak force (N) †	1535.8 ± 216.8 (1428.0–1643.6)	1575.1 ± 285.5 (1433.1–1717.1)	1618.4 ± 213.8 (1512.1–1724.7)	1615.1 ± 254.8 (1488.4–1741.8)
	RFD_max_ (N.m.s^−1^)	6.8 ± 1.1 (6.1–7.5)	6.8 ± 1.7 (6.1–7.4)	6.4 ± 1.3 (5.4–6.9)	6.7 ± 1.4 (6.2–7.2)
	Force _50 ms_ (% MVC)	10.1 ± 2.8 (8.6–11.7)	10.2 ± 2.8 (8.5–11.9)	11.2 ± 4.2 (8.8–13.5)	9.7 ± 2.9 (8.5–11.1)
	Force _100 ms_ (% MVC)	26.9 ± 6.7 (23.3–30.7)	26.9 ± 7.7 (22.3–31.6)	25.9 ± 7.8 (21.6–30.2)	24.7 ± 8.1 (21.3–28.1)
	Force _150 ms_ (% MVC)	49.4 ± 11.1 (41.3–51.0)	47.4 ± 11.5 (41.6–53.2)	45.7 ± 12.7 (36.9–49.3)	44.6 ± 12.5 (40.1–49.1)
	Force _200 ms_ (% MVC)	63.1 ± 9.1 (56.5–65.9)	62.6 ± 12.2 (56.4–68.9)	59.6 ± 12.7 (50.7–64.5)	60.4 ± 13.4 (55.2–65.7)
Countermovement squat jump	Jump height (cm) ‡	37.6 ± 5.9 (34.7–40.6)	37.7 ± 5.7 (36.2–39.3)	37.1 ± 5.9 (34.2–40.1)	38.6 ± 6.7 (37.7–39.5)
	Peak force (N) ‡	1010.9 ± 150.0 (936.3–1085.5)	1023.5 ± 198.7 (936.3–1085.5)	1071.8 ± 213.9 (965.4–1178.2)	1038.9 ± 150.0 (965.4–1178.2)
	Dynamic strength index	0.67 ± 0.11 (0.59–0.71)	0.66 ± 0.13 (0.62–0.72)	0.67 ± 0.16 (0.57–0.77)	0.65 ± 0.09 (0.59–0.76)
Squat jump	Jump height (cm)	34.0 ± 5.4 (30.5–36.8)	35.0 ± 5.2 (33.5–36.6)	34.1 ± 5.1 (31.1–37.1)	34.8 ± 5.5 (33.9–35.9)
	Peak force (N) ‡	928.9 ± 177.8 (825.1–1039.1)	895.5 ± 125.9 (857.1–933.9)	944.1 ± 200.7 (855.0–1078.9)	951.6 ± 179.8 (917.8–985.3)
	Dynamic strength index	0.61 ± 0.11 (0.53–0.67)	0.58 ± 0.12 (0.53–0.63	0.59 ± 0.16 (0.52–0.69)	0.60 ± 0.11 (0.56–0.63)

Values are mean ± SD. Abbreviations: RFD_max_, maximum rate of force development; MVC, maximum voluntary contraction; Force _50 ms_, force produced at 50 ms of isometric mid-thigh pull normalized to maximum voluntary contraction; Force _100 ms_, force produced at 100 ms of isometric mid-thigh pull normalized to maximum voluntary contraction; Force _150 ms_, force produced at 150 ms of isometric mid-thigh pull normalized to maximum voluntary contraction; Force _200 ms_, force produced at 200 ms of isometric mid-thigh pull normalized to maximum voluntary contraction. † Condition main effect (ANOVA: *p* = 0.01 at pre-intervention). ‡ Condition main effect (ANCOVA: *p* = 0.03, *p* = 0.04 and *p* = 0.02 at post-intervention, respectively).

**Table 4:** Changes in indices of muscle strength (upper limb) after four weeks of time-restricted eating (TRE) and habitual diet (non-TRE).

		TRE		Non-TRE	
		Pre-Intervention	Post-Intervention	Pre-Intervention	Post-Intervention
*Isometric Strength—Bench Press*	Peak Force (N)	820.3 ± 162.9 (739.3–901.3)	797.5 ± 105.7 (770.7–824.3)	799.6 ± 102.0 (748.9–850.4)	809.6 ± 94.6 (772.9–846.3)
	RFD_max_ (N.m.s^−1^) ‡	4.5 ± 1.2 (3.9–5.1)	4.4 ± 1.3 (4.1–4.8)	4.3 ± 0.8 (3.9–4.7)	4.8 ± 0.6 (4.5–5.0)
	Force _50 ms_ (% MVC)	15.5 ± 6.1 (12.5–18.5)	17.7 ± 7.3 (13.9–21.6)	16.5 ± 5.8 (13.6–19.4)	18.1 ± 2.9 (17.1–20.6)
	Force _100 ms_ (% MVC) *	37.4 ± 13.6 (30.6–44.2)	46.5 ± 14.9 (37.8–55.1)	41.1 ± 13.8 (34.2–48.0)	47.0 ± 6.5 (43.5–50.5)
	Force _150 ms_ (% MVC) *	54.8 ± 17.8 (45.9–63.7)	67.7 ± 11.7 (60.6–74.9)	61.2 ± 18.8 (51.8–70.5)	68.3 ± 7.9 (63.7–72.9)
	Force _200 ms_ (% MVC) *	62.1 ± 18.7 (52.8–71.4)	74.7 ± 14.7 (65.7–83.7)	69.5 ± 19.5 (59.8–79.2)	77.2 ± 7.8 (72.3–82.2)
*Ballistic Strength* *—Bench press throw*	Peak Force (N) §	639.8 ± 87.6 (596.2–683.3)	652.3 ± 93.6 (630.8–673.8)	641.9 ± 87.1 (598.5–685.2)	547.4 ± 134.0 (479.8–615.0)
	Mean Velocity (m.s^−1^)	0.88 ± 0.13 (0.80–0.93)	0.89 ± 0.09 (0.85–0.92)	0.89 ± 0.13 (0.83–0.96)	0.89 ± 0.10 (0.85–0.94)
	Mean power (W)	500.4 ± 175.0 (413.3–587.4)	541.9 ± 194.1 (477.4–606.4)	517.3 ± 161.7 (436.9–597.7)	559.6 ± 199.9 (497.9–621.3)
	Dynamic strength index §	0.79 ± 0.13 (0.74–0.86)	0.82 ± 0.11 (0.80–0.85)	0.81 ± 0.11 (0.76–0.87)	0.68 ± 0.18 (0.60–0.77)

Values are mean ± SD. Abbreviations: RFD_max_, maximum rate of force development; MVC, maximum voluntary contraction; Force _50 ms_, force produced at 50 ms of isometric bench press normalized to maximum voluntary contraction; Force _100 ms_, force produced at 100 ms of isometric bench press normalized to maximum voluntary contraction; Force _150 ms_, force produced at 150 ms of isometric bench press normalized to maximum voluntary contraction; Force _200 ms_, force produced at 200 ms of isometric bench press normalized to maximum voluntary contraction. * Time main effect (ANOVA: *p* = 0.01, *p* = 0.01 and *p* = 0.04 from pre- to post-intervention, respectively). ‡ Condition main effect (ANCOVA: *p* = 0.04 at post-intervention). § Condition-by-time main effect (ANOVA: *p* = 0.005 and *p* = 0.001 from pre- to post-intervention, respectively).

## Data Availability

The datasets used and/or analyzed during the current study are available from the corresponding author on reasonable request.
